# GPs’ perspectives on acne management in primary care: a qualitative interview study

**DOI:** 10.3399/bjgp20X713873

**Published:** 2020-12-01

**Authors:** Duncan Platt, Ingrid Muller, Anicka Sufraz, Paul Little, Miriam Santer

**Affiliations:** Primary Care Research Centre, University of Southampton, Southampton.; Primary Care Research Centre, University of Southampton, Southampton.; GKT School of Medical Education, King’s College London, London.; Primary Care Research Centre, University of Southampton, Southampton.; Primary Care Research Centre, University of Southampton, Southampton.

**Keywords:** acne vulgaris, antibiotics, GPs’ perspectives, primary care, qualitative research

## Abstract

**Background:**

Acne is a common skin condition, affecting most adolescents at some point. While guidelines recommend topical treatments first-line, long courses of oral antibiotics are commonly prescribed.

**Aim:**

To explore GPs’ perspectives on managing acne.

**Design and setting:**

Qualitative interview study with GPs in South West England.

**Method:**

GPs were invited to participate via existing email lists used by GP educators to disseminate information. Purposive sampling was used to recruit a range of participants by sex, number of years in practice, and whether their practice was rural or urban. Semi-structured telephone interviews followed an interview guide and were audiorecorded and transcribed. Data were explored using inductive thematic analysis facilitated by NVivo software (version 11).

**Results:**

A total of 102 GPs were invited, of whom 20 participated. Analysis revealed uncertainties regarding topical treatments, particularly around available products, challenges regarding side effects, and acceptability of topical treatments. GPs generally either perceived topical treatments to be less effective than oral antibiotics or perceived pressure from patients to prescribe oral antibiotics due to patients’ views of topical treatments being ineffective. GPs described a familiarity with prescribing oral antibiotics and expressed little concern about antimicrobial stewardship in the context of acne. Some seemed unaware of guidance suggesting that antibiotic use in acne should not exceed 3 months, while others spoke about avoiding difficult conversations with patients regarding discontinuation of antibiotics.

**Conclusion:**

GPs expressed uncertainty about the use of topical treatments for acne and either felt that treatments were of low effectiveness or perceived pressure from patients to prescribe oral antibiotics.

## INTRODUCTION

Acne vulgaris (hereafter ‘acne’) is a very common chronic skin condition.^1^ Almost all teenagers are affected to some degree, with between 15% and 20% having moderate or severe disease.^2^ Acne can continue into adulthood, affecting >40% of individuals in their 30s.^3^^,^^4^ The psychological impact can be significant, including an increased risk of depression and suicide.^2^

UK, European, and US guidelines indicate that non-antibiotic topical treatments, such as benzoyl peroxide, or a topical retinoid/adapalene, should generally be used first-line for mild acne, followed by second-line topical treatments, including combination products containing antibiotics.^5^^–^^7^ Adherence to topical treatments can be poor because they may cause initial skin irritation.^2^ Clinician advice regarding application, for instance, advising patients to start applying treatments every other day and then to increase the frequency, is thought to improve outcomes.^2^^,^^8^

Systemic treatments include oral antibiotics and, for women, combined oral contraception or co-cyprindiol.^5^ Oral antibiotics should be used in patients with moderate acne who have failed to respond to topical treatment.^5^ When oral antibiotics are used, this should be alongside topical non-antibiotics, and they should be used for a maximum period of 3 months to reduce the risk of antibiotic resistance.^5^^–^^7^ There have been few trials comparing topical therapies with oral antibiotics, although the available evidence suggests they may be similarly effective.^9^^,^^10^

There are growing concerns about antibiotic resistance in the treatment of acne.^11^^,^^12^ Topical antibiotics can cause the emergence of resistant bacteria at skin sites.^12^^,^^13^ Oral antibiotics can lead to resistant strains, both on the skin and at other body sites including the gut.^11^ Antibiotic prescribing for skin conditions represents a large proportion of antibiotic use among young people.^14^ Although rates of prescribing of penicillins among young people are falling, prescriptions for macrolides and tetracyclines continue to rise.^14^ While further investigations of these trends are needed, much of this prescribing is likely to be for acne (M Lown, personal communication, 2020).

Most acne treatment in the UK is provided in general practice.^15^ Despite this, relatively little research has studied the management of acne in UK primary care. A cohort study analysed consultations and prescribing for acne using the Clinical Practice Research Datalink.^16^ It found that the most common prescription given at the initial acne consultation was an oral antibiotic alone (34%), closely followed by topical antibiotics (32%).^16^ This is in line with earlier research, which found that the most commonly prescribed treatment for acne was oral antibiotics.^15^

**Table:** How this fits in

Despite rising concerns about antibiotic resistance, long courses of oral antibiotics remain the most widely prescribed treatment for acne in the UK. Guidelines advise that topical treatments should be used first-line for acne and evidence suggests their effectiveness is similar to that of oral antibiotics. GPs need support in prescribing and promoting the effective use of topical treatments for acne; for instance, through easy access to high-quality information to give patients. Patients need to know that topical treatments work well for acne if used daily for 4–6 weeks, and should be given advice about how to prevent side effects such as skin irritation.

While the reasons for this are unclear, the data suggest significant overprescribing of antibiotics and underuse of alternative non-antibiotic strategies. Research in the US has also suggested that long courses of oral antibiotics are common.^17^ The aim of the present study was to explore GPs’ perspectives of managing acne, and the facilitators and barriers to prescribing treatments including oral antibiotics and topical treatments.

## METHOD

### Recruitment

GPs were invited to participate by using existing email lists used by GP educators in South West England to disseminate information and advertise education events. Purposive sampling was used to recruit a range of participants by sex, years in practice, and whether their practice was rural or urban to seek diverse views. One author noted participants’ characteristics as the interviews progressed and, for the later interviews, sent reminder emails selectively to GPs in under-represented groups, particularly those who had been in practice for longer or who were working in more urban areas.

### Interviews

One author conducted semi-structured interviews by telephone using an interview guide based on the available literature^2^^,^^18^ and study aims (Box 1). The guide was developed and piloted before commencing the interviews and minor revisions to the wording were made following the first two interviews. Written consent was obtained, and a reimbursement of 50 GBP in shopping vouchers was offered. Interviews were audiorecorded, transcribed verbatim, and checked.

**Box 1.** Interview guide questions and prompts

How do you feel about managing acne as a GP? – Are there any particular challenges? What are your thoughts on why patients with acne come to see you? How do you assess patients with acne? – What questions do you ask? – What assessment do you make when you look at the appearance of the skin? – Anything that is particularly important? What are your general thoughts about treatment options for acne? – How do you choose which to prescribe? How are your thoughts on topical treatments for acne? – Please specify treatment names – Any that you particularly prefer to prescribe? Why? – Any that you particularly avoid? Why? – What advice do you give? – How do you think patients feel about using topical treatments? (Please specify the treatment’s name) What are your thoughts on prescribing oral antibiotics for acne? – How do you think patients feel about taking antibiotics for acne? – What advice do you give? What are your thoughts on prescribing the combined contraceptive pill for acne? – How do you think patients feel about using the contraceptive pill for acne? – What advice do you give? What factors are important for you in choosing to refer patients with acne? What other advice do you routinely offer patients with acne? Are there any supporting materials either written or online that you use to: – Support your management of acne? – Support your patients’ management of their acne? Are there any supporting materials not currently provided that you feel could: – Support your management of acne? – Support your patients’ management of their acne?

### Analysis

Interview data were explored using an inductive thematic analysis.^19^ One author read and re-read transcripts to become familiar with the data. Codes were inductively derived from the data before being grouped together to form an initial coding frame. Codes, themes, and subthemes were defined, discussed, and iteratively developed by four members of the research team. One author applied the coding frame to all transcripts and another applied the coding frame to three transcripts and then compared interpretations. NVivo software (version 11) was used to aid data organisation and coding. Data collection continued until saturation of the main themes was reached.

## RESULTS

A total of 102 GPs were invited to take part, of whom 21 agreed, and 20 were interviewed. One GP was unable to be interviewed because of a conflicting work arrangement and was not able to reschedule. Semi-structured telephone interviews were carried out between February 2018 and April 2019, with interviews lasting between 15–37 mins. Participant characteristics are shown in Table 1. Only one of the GPs interviewed had a special interest in dermatology.

**Table 1.** Participant characteristics

**Variable**	**Interviewees (N = 20), *n* (%)**
Female	10 (50)

Years in practice	
≤10	9 (45)
11–20	6 (30)
>20	5 (25)

Dermatology GPSI (current or previous)	1 (5)

Practice location, urban	14 (70)

GPwER = GP with extended role.

### Key themes

Thematic analysis of the interview data relating to prescribing of topical treatments and oral antibiotics established four key themes:

- uncertainties regarding prescribing topical treatments;
- perceptions regarding side effects and acceptability to patients of topical treatments;
- GPs’ perceptions of lower effectiveness of topical treatments compared with oral antibiotics; and
- familiarity with prescribing oral antibiotics and low concern about prolonged courses for acne.

Each of these themes are described in detail below, illustrated with selected quotes.

### Uncertainties regarding prescribing topical treatments

Despite topical treatments being first-line for mild and moderate acne, interviewees expressed uncertainty about the different options available, whether on prescription or over the counter, and how to choose between them. Frequently, they struggled to recall names or constituents of the products during the interviews:

‘I think it can be a bit confusing because, particularly on the topical agents, there’s a lot of products that cross over and are similar, and it can get a bit muddled between all the brands and things.’

(GP6, male [M], 11–20 years in practice)

Many GPs reported choosing topical treatments based on familiarity and habit:

‘I think that is one of those areas, again, because it’s — there are so many choices of what you can do and I got very familiar with what I was familiar with.’

(GP13, M, >20 years in practice)

Interviewees were most familiar with prescribing topical benzoyl peroxide and topical antibiotics. In contrast, they had less confidence about prescribing topical retinoids:

*‘I’ve probably only used it* [adapalene] *a handful of times, but actually the handful of times I’ve used it, it has been quite successful. I suppose the reason why I don’t use it more often is probably just my own confidence about using it*. *’*

(GP15, female [F], ≤10 years in practice)

### Perceptions regarding side effects and acceptability to patients of topical treatments

GP interviewees often associated topical treatments with frequent localised side effects, which were viewed as problematic for patients:

‘They’ve got lots of side effects; the drying, the redness, the stained clothes, the bleaching of the sheets; there’s lots of complications that makes them socially not very usable.’

(GP5, F, >20 years in practice)

Most GPs interviewed said that they felt able to offer patients basic advice regarding applying topical treatments and managing side effects. However, they did not describe routinely signposting patients to other resources, written or online, to support the patient’s use of topical treatments. Some expressed scepticism that commonly available written formats were suitable:

*‘That’s something I could probably learn from and use would be online guides or written guides, but I don’t tend to use those* . *’*

(GP1, M, ≤10 years in practice)

*‘I know it’s all available as medicine information inside the pack, but, you know, realistically, when you’ve got a young person, they probably aren’t going to be reading all that bumph* ... *some slightly more modern sort of stuff on social media, more targeted at young people, might not be a bad idea.’*

(GP15, F, ≤10 years in practice)

### GPs’ perceptions of lower effectiveness of topical treatments compared with oral antibiotics

GP interviewees commonly said they thought topical treatments were less effective than oral treatments, were predominantly useful only for mild acne, and that patients who were consulting the GP would not wish to be *‘completely fobbed off’* (GP18, M, >20 years in practice) with a topical treatment:

‘And generally, to be fair, patients that come in to see us have already tried a lot of the topical stuff on their own, albeit they probably have only got benzoyl peroxide in them, because that’s pretty much, as far as I’m aware, all you can really buy over the counter, but generally the severity that bothers most people’s skin — is severe enough that you probably need more than just topical stuff.’

(GP11, F, ≤10 years in practice)

In contrast, oral antibiotics were viewed as more effective and quicker acting:

*‘So someone who has a really good reason to reduce the lesions as quickly as they can, that might have mild, moderate* [acne] *, I might go with oral antibiotics quicker than someone who isn’t that bothered and has localised disease, and is happy to try a topical treatment.’*

(GP13, M, >20 years in practice)

‘I don’t know what percentage I would say, but definitely 50% of patients, probably more like 70, 75%, you know, are going to — normally tend to have success on antibiotics.’

(GP15, F, ≤10 years in practice)

While some GPs perceived that oral treatments were more effective than topical treatments, others perceived that topical treatments were as effective but said they felt a pressure from patients to prescribe oral rather than topical treatments, based on a belief that they are more effective:

*‘So I would say — that it generally should be managed with topicals but we quite often go to oral because that’s what — by the time they get to us, that’s what people want. They think that is more effective, even if that’s not the case — and it’s easier for them* . *’*

(GP2, F, 11–20 years in practice)

Concerns around difficulty with treatment adherence and side effects for topical treatments, as described above, also arose in discussions around effectiveness. One participant also linked this difficulty with appropriate use to a perception of topical treatments as being ineffective:

‘A tablet you can just swallow and that’s it done, whereas with a cream, you’ve got to apply it and apply it appropriately and let it soak in and so on. So I guess it’s a bit more complicated a regimen, so it perhaps doesn’t feel quite so effective.’

(GP10, M, ≤10 years in practice)

### Familiarity with oral antibiotics and low concerns about prolonged courses for acne

In contrast with uncertainties expressed around prescribing topical treatments, many GPs reported feeling relaxed prescribing oral antibiotics for acne:

*‘I’m really happy to and I think probably, again, rightly or wrongly, I have a low threshold to start them on oral antibiotics. I think I just feel for them. I just think if you’ve got a whole load of spots on your face, I can completely appreciate that it’s a nightmare* . *’*

(GP16, F, ≤10 years in practice)

Despite guidelines recommending that oral antibiotics should be used for no longer than 3 months,^5^ interviewees were generally either not aware of this or viewed prolonged courses to be necessary or difficult to avoid if patients requested this:

*‘Minimum 6 months would not be unusual, particularly anything above mild* [acne] *which you’re not going to use oral antibiotics for anyway, whereas the moderate to moderate/severe is going to take about a year.’*

(GP13, M, >20 years in practice)

*‘You know, when you put them on it, you put them on* [antibiotics] *— I know you’re meant to review it sort of every 3 months or so, but actually a lot of the time I think we tend to go — okay, you’re doing fine, let’s put it on repeat and we’ll see you in 6 months.’*

(GP16, F, ≤10 years in practice)

A frequently reported challenge was that of stopping oral antibiotics in patients who have taken them for a long time and where conversations about stopping were viewed as ‘difficult’:

‘I think it’s often difficult, because actually, usually when you go through repeat prescriptions, often people are on antibiotics, I find, for years, for skin, whether it’s for acne or even rosacea. Actually, usually they’re put on repeat and I think the conversation is often avoided, about stopping it. And I find it difficult, as someone who’s suffered with their skin, I think it’s — it is difficult to have that conversation with people.’

(GP11, F, ≤10 years in practice)

As expected, GP interviewees were aware of concerns about antibiotic resistance in general; however, it was striking that most viewed it as a low priority in the context of antibiotic prescribing in acne:

‘Am I worried about it? In truth, probably not, actually. I mean I’m conscious of it, so I’m not going to treat people, you know, for no reason, with an antibiotic, but I think if they’ve got to the point where their acne is causing significant problems and they’ve not responded to topical treatments, then, you know, or is severe enough, I don’t have any concerns about prescribing long-term antibiotics.’

(GP6, M, 11–20 years in practice)

‘Concerns, not really. I mean we’ve been prescribing all antibiotics for acne for a long time and I’m not aware that there’s any major issue associated with it. Although in some ways that does seem a bit bizarre because we look at our antibiotic prescribing elsewhere and of course we’re really jittery about it and I guess there’s a bit of me that becomes increasingly concerned about the impact of antibiotics on your biome.’

(GP18, M, >20 years in practice)

## DISCUSSION

### Summary

GPs interviewed in this study described challenges with using topical treatments in terms of uncertainty about the topical treatments available, difficulties for patients with adherence, and side effects. GPs often perceived topical treatments to be of low effectiveness compared with oral antibiotics, or spoke about pressure from patients to prescribe oral treatments because patients perceived them as being more effective. GPs described familiarity with and relatively low concern when prescribing oral antibiotics, including for longer durations than the 3 months recommended in UK guidance.^5^ They described conversations about withdrawing antibiotics as being potentially difficult and sensitive.

### Strengths and limitations

The interviewees were purposively sampled to ensure a range of characteristics including sex, years in practice, and practice location. GPs were sampled through educational and research lists in South West England and only 20% agreed to take part. It seems unlikely that a broader sample of GPs would have revealed different views, given that data saturation was achieved. However, it is possible that our recruitment strategy may have led to inclusion of participants with relatively high awareness of recent educational or research developments. There is the potential when conducting interviews that the views expressed do not fully represent everyday practice, and as the interviewer was a GP, it is possible that this could have contributed to interviewees offering ‘socially desirable’ responses. Conversely, others have found that professionals interviewing each other can lead to *‘richer and more personal accounts of attitudes and behaviour’*.^20^ The interviewer found that the participants were willing to share a diverse range of candid views. Furthermore, the findings regarding the relatively low use of topical treatments compared with oral antibiotics for acne is in line with observed prescribing data.^16^

### Comparison with existing literature

Despite many studies that have explored GPs’ perceptions about prescribing antibiotics for acute illness, the authors are not aware of any previous research exploring GPs’ perspectives on antibiotic prescribing for acne, despite this being a major cause of antibiotic use.

Qualitative research into patients with acne has similarly found a perception of low effectiveness of topical treatments in comparison with oral antibiotics.^18^ However, to the authors’ knowledge, the present study is the first to show that many GPs also hold this view and to explore the implications of GPs’ perceptions of patient expectations about antibiotic prescribing for acne. One previous qualitative study of GPs’ views of acne management found that GPs perceived patients’ non-adherence to topical treatments as a barrier to treatment success but that they rarely signposted patients to written information sources.^21^

The current study’s findings are in line with previous research in the UK and US, which found that oral antibiotics are widely used for acne,^16^^,^^17^ and that average courses are, in practice, often longer than 3 months.^17^

The findings echo previous literature on doctors’ behaviours around prescribing and referral, where perceived pressure from patients is a predictor of prescribing behaviours.^22^^,^^23^ Similarly, when prescribing antibiotics for other conditions, doctors’ perceptions of patient expectations for antibiotics is a predictor of prescribing, although doctors often overestimate patients’ expectations for receiving an antibiotic.^24^

### Implications for practice

There is a need to promote alternative management strategies and clearer guidance to address the high levels of antibiotic prescribing for acne. Although most guidelines state that the use of antibiotics for acne should generally be limited to 3 months,^5^^–^^7^ unfortunately other sources of reference, such as the *British National Formulary*, continue to suggest longer courses may be needed, such that some confusion and lack of adherence to guidelines among prescribers is unsurprising.

A greater understanding of GPs’ perspectives about acne management provides potential behavioural targets for a training intervention to reduce antibiotic prescribing in acne. Interventions to reduce antibiotic prescribing in primary care for respiratory tract infections have been effective in a number of countries,^25^ and similar interventions may be effective in reducing antibiotic prescribing in acne. Promoting the use of alternatives to antibiotics in acne includes effective communication with patients about how to mitigate against side effects of topical treatments in mild or moderate acne, and widening access to timely prescribing of oral isotretinoin for severe acne.
